# Response Surface Methodology Application for Bacteriophage–Antibiotic Antibiofilm Activity Optimization

**DOI:** 10.3390/microorganisms11092352

**Published:** 2023-09-20

**Authors:** Bartłomiej Grygorcewicz, Marta Gliźniewicz, Patrycja Olszewska, Dominika Miłek, Artur Czajkowski, Natalia Serwin, Elżbieta Cecerska-Heryć, Rafał Rakoczy

**Affiliations:** 1 Faculty of Pharmacy, Medical Biotechnology and Laboratory Medicine, Pomeranian Medical University, Powstańców Wielkopolskich 72, 70-111 Szczecin, Poland; marta.glizniewicz@pum.edu.pl (M.G.); pati.olszewska@onet.pl (P.O.); dominikamilek@icloud.com (D.M.); artcza11@icloud.com (A.C.); natalia.serwin@pu.edu.pl (N.S.); elzbieta.cecerska.heryc@pum.edu.pl (E.C.-H.); 2 Department of Chemical and Process Engineering, Faculty of Chemical Technology and Engineering, West Pomeranian University of Technology in Szczecin, Piastów Ave. 42, 71-065 Szczecin, Poland; rafal.rakoczy@zut.edu.pl

**Keywords:** antimicrobials, biofilm, phages, mathematical modeling

## Abstract

Phage–antibiotic combination-based protocols are presently under heightened investigation. This paradigm extends to engagements with bacterial biofilms, necessitating novel computational approaches to comprehensively characterize and optimize the outcomes achievable via these combinations. This study aimed to explore the Response Surface Methodology (RSM) in optimizing the antibiofilm activity of bacteriophage–antibiotic combinations. We employ a combination of antibiotics (gentamicin, meropenem, amikacin, ceftazidime, fosfomycin, imipenem, and colistin) alongside the bacteriophage vB_AbaP_AGC01 to combat *Acinetobacter baumannii* biofilm. Based on the conducted biofilm challenge assays analyzed using the RSM, the optimal points of antibiofilm activity efficacy were effectively selected by applying this methodology, enabling the quantifiable mathematical representations. Subsequent optimization showed the synergistic potential of the anti-biofilm that arises when antibiotics are judiciously combined with the AGC01 bacteriophage, reducing biofilm biomass by up to 80% depending on the antibiotic used. The data suggest that the phage–imipenem combination demonstrates the highest efficacy, with an 88.74% reduction. Notably, the lower concentrations characterized by a high maximum reduction in biofilm biomass were observed in the phage–amikacin combination at *c_A_* = 0.00195 and *c_P_* = 0.38 as the option that required minimum resources. It is worth noting that only gentamicin antagonism between the phage and the antibiotic was detected.

## 1. Introduction

It is estimated that approximately 65% of bacterial infections are related to biofilms, and to make matters worse, bacteria in the form of biofilms pose a severe threat due to their many times greater tolerance to antibiotics [1,2]. Bacteriophages, or phages for short, are viruses that specifically infect their host bacteria and pose promising treatment options for biofilm-related infections caused by antibiotic-resistant bacteria [3,4,5,6]. The best therapeutic approach focuses on combining phages and antibiotics that can interact synergistically. This phenomenon is attributed to bacteriophages and antibiotics synergy (PAS) [4,7]. Combining antibiotic therapy with phage therapy using antibiotics that have a synergistic effect of antibiotics can increase the effectiveness of treatments [7,8,9]. Regrettably, the primary challenge in developing the combined treatment lies in the meticulous selection of bacteriophages and antibiotics that can exhibit synergistic effects. This selection is of utmost importance considering contemporary research, which has demonstrated that the synergistic action observed in these combined treatment modalities implies the potential PAS effects [3,10]. The growing interest in bacteriophages and antibiotics’ combined use poses new challenges regarding this phenomenon’s accurate description [3,8,11,12].

The synergistic effect of the concentrations of various types of antibiotics and phages may be evaluated using the Response Surface Methodology (RMS), a widely used mathematical tool for optimization [13]. Based on the Design of Experiments (DoE), this method is also used to optimize the effect of process variables [14]. The main aim of this method is to determine the interaction between the independent variables, alone or in combination, in the form of a mathematical model [15]. This model generates an accurate mathematical description of the overall process and may be used to analyze the effects of the independent variables. The RMS method describes the interactive effects of process variables on the process in the form of a graphical representation [14,16]. To obtain the response surface and the second-order polynomial models in RMS, the fractional designs (the Central Composite Design (CCD) or Box–Behnken Design (BBD)) are applied [16]. The application of RSM is allowed to select the best experimental conditions requiring the lowest number of experiments to obtain appropriate results [17,18].

The Response Surface Methodology (RSM) is an experimental technique used for the optimization and analysis of processes, aiming to identify optimal parameters that lead to achieving the desired outcome of the process [19]. This method assumes a response surface, which describes the relationship between various experimental factors and the process response (e.g., product quality, efficiency, costs, etc.). This surface is typically approximated via polynomials or other mathematical functions that allow for predicting response values for different combinations of factors. Moreover, the interaction effect between different parameters can be analyzed systemically with fewer required experiments [20].

The Response Surface Method (RMS) is based on experimental techniques used to investigate the relationship between experimental factors and measured responses. The RMS can obtain the relation between the independent and dependent variables or variables [21]. From the mathematical point of view, this relationship is expressed in the following form [22]:

(1) $$y=\beta_{0}+\sum_{i=1}^{k}\beta_{i}x_{i}+\sum_{i=1}^{k}\beta_{ii}x_{i}^{2}+\sum_{1\leq i\leq j}^{k}\beta_{ij}x_{i}x_{j}+\varepsilon$$

where

*k*—is the number of input variables;
*x_i_*—is the input variable;
*y*—is the response (output variable);
*β*_0_—is the constant term;
*β_i_*—is the coefficient of the linear variables;
*β_ii_*—is the coefficient of the quadratic parameter;
*β_ij_*—is the interaction coefficient between the input variables;
*ε*—is the error associated with the experiments.

The Response Surface Methodology offers numerous advantages for optimizing antibiofilm and antimicrobial activities. Singh et al. (2023) describe the RSM-driven design and synthesis of carbohydrate/sulfated polysaccharide-capped silver nanocomposites, resulting in the precise control over their properties and enhancing antibiofilm characteristics. Moreover, RSM aided in characterizing nanocomposites and optimizing stability. Antibiofilm studies showcased the potency of these nanocomposites, especially in eradicating *Pseudomonas aeruginosa* biofilms, highlighting RSM’s role in enhancing their antibiofilm properties [23]. Gharbani et al. (2023) demonstrated RSM’s ability to assess the synergistic antibacterial effect of natural compounds, offering an eco-friendly alternative to chemical preservatives. RSM revealed significant synergies between *Punica granatum* L. and Areca nut extracts, particularly against *Escherichia coli* [24]. The RSM optimization enhanced the antimicrobial activity of surfactin and polylysine against *Salmonella* Enteritidis, providing critical insights into their sensitivity and effectiveness in reducing bacterial load [25]. Overall, RSM is a valuable tool for understanding, fine-tuning, and developing effective antimicrobial and antibiofilm solutions.

This study aims to utilize Response Surface Methodology for the first time in bacteriophage–antibiotic combinations to optimize their antibiofilm activity. This research seeks to assess the impact of bacteriophages and antibiotics on combatting bacterial biofilms and to identify the optimal conditions that maximize their synergistic antibiofilm efficacy.

## 2. Materials and Methods

### 2.1. Bacterial Strain and Bacteriophage Used

*Acinetobacter baumannii* clinical isolate (PUM45202) identified by VITEK^®^2 compact (bioMérieux Inc., Durham, NC, USA) was used in this study. The bacterial strain was stored at −80 °C before use and was grown on blood agar (bioMérieux Inc., Durham, NC, USA) for 18–24 h at 37 °C to revitalize. This strain was described before by Grygorcewicz et al. (2022) [26]. CFU of *Acinetobacter baumannii* was analyzed with standard plating methods on LB medium. The bacteriophage vB_AbaP_AGC01 was used in this study. Used bacteriophage was previously characterized by Grygorcewicz et al. (2020) [8]. Bacteriophage was propagated as described before using LB medium (Miller modification, Merck KgaA, Darmstadt, Germany) at 37 °C with shacking 160 rpm for 4 h. After that, phage lysate was centrifuged and filtered through a 0.22 μm polyethersulfone membrane syringe filter. The standard double-layer agar method assessed phage viable particles as described before [27].

### 2.2. Antibiotics Used in This Study

In this study, gentamicin (cat. No. G1397), meropenem (cat. No. 1392454), amikacin (cat. No. PHR1860), ceftazidime (cat. No. C3809), imipenem (cat. No. I0090000), and colistin (cat. No. 1148001) were used to analyze the MICs and phage antibiotic combination activity. All antibiotics used in this study were acquired from Merck KgaA, Darmstadt, Germany.

### 2.3. Antibiofilm Activity

To produce biofilm, the *A. baumannii* strain (10^5^ CFU/mL) was inoculated in TSB medium (Merck KgaA, Darmstadt, Germany) and incubated for 48 h at 37 °C with medium replacement every 12 h by removing the medium and double washing it with PBS buffer. After incubation, the biofilm was rinsed five times with PBS buffer and immersed in a TSB medium prepared by combining an antibiotic (in concentration ranging from 0 to 1024 µg/mL) and phages (in concentration from 10^3^ to 10^8^ PFU/mL). The inoculated plates were incubated for 8 h at 37 °C. The biofilm biomass assay based on crystal violet biomass staining is presented elsewhere [27,28]. The bacterial culture was removed, and the formed biofilm was rinsed with PBS buffer and fixed with methanol for 10 min. Plates were dried at room temperature, stained with crystal violet solution (1%, Merck KgaA, Darmstadt, Germany), and incubated for 10 min. After incubation, crystal violet was removed, and plates were washed in tap water and dried. Glacial acetic acid (33%, Merck KgaA, Darmstadt, Germany) and methanol (2:8) were used to resolubilize crystal violet. The absorbance was measured at 595 nm using a BioTek Synergy H1 plate reader.

### 2.4. Data Normalization

It should be noticed that the values of antibiotic concentration and phage concentration are normalized (Table 1) as follows:

(2) $$c_{A}=\frac{c_{A\_var}}{c_{A\_max}}$$

And

(3) $$c_{P}=\frac{c_{P\_var}}{c_{P\_max}}$$

where

*c_A_var_*  —is the value of antibiotic concentration (this value was varied in the range of 0 to 1024), µg/mL;
*c_A_max_*  —is the maximum value of antibiotic concentration (this value is equal to 1024), µg/mL;
*c_P_var_*  —is the value of phage concentration (this value was varied in the range of 0 to 10^8^), PFU/mL;
*c_P_max_*  —is the maximum value of phage concentration (this value is equal to 10^8^), PFU/mL.

**Table 1:** Results of data normalization.

Antibiotic Concentration	Normalized Antibiotic Concentration	Phage Concentration	Normalized Phage Concentration
[µg/mL]	*c_A_* [-]	[PFU/mL]	*c_P_* [-]
1024	1	10^8^	1
512	0.5	10^7^	0.1
256	0.25	10^6^	0.01
128	0.125	10^5^	0.001
64	0.0625	10^4^	0.0001
32	0.03125	10^3^	0.00001
16	0.015625	0	0
8	0.007813		
4	0.003906		
2	0.001953		
1	0.000977		
0	0		

### 2.5. Statistical Analysis

To assess the impact of independent variables (antibiotics and phage concentrations) on the percentage reduction (% reduction), we use the Statistica 13.3 software (TIBCO Software Inc., Palo Alto, CA, USA). To determine the optimal levels of antibiotics and phage concentrations for enhancing the proposed phage-assisted antibiotic therapy, we employed Response Surface Methodology (RSM) based on the Central Composite Design (CCD).

The behavior of the phage-assisted antibiotic therapy was elucidated using the following quadratic equation. We tested the significance of the process parameters and their interactions via ANOVA at a 95% confidence level. The overall model significance was assessed using Fisher’s F-test and associated probability. To evaluate the quality of the polynomial equation fit, we utilized the coefficient of determination (R2). To illustrate the relationship between the response (% reduction) and the experimental levels of independent variables, we created three-dimensional surface plots. These plots help visualize the effects of antibiotics and phage concentrations on the % reduction.

We used the Pareto plot to identify the most influential factors the Pareto plot was used [29]. This plot displays which effects (antibiotics or phage concentration) and two-factor interactions (between antibiotics and phage concentrations) are statistically significant at a 5% significance level.

## 3. Results

To achieve the optimal response (maximum % reduction of biofilm biomass), the interaction between two variables (phage concentration and antibiotics concentration) is depicted using response surface graphs and Pareto charts. The graphical presentations of the Pareto results were prepared to demonstrate the influence of variables on the response.

The most crucial variable is determined by selecting the variables that exhibit the most excellent standardized effect on most responses in these graphs. The length of the bar is proportional to the standardized product. The symbols “L” and “Q” denote the linear and quadratic effects in Equation (1), respectively.

**Figure 1:**
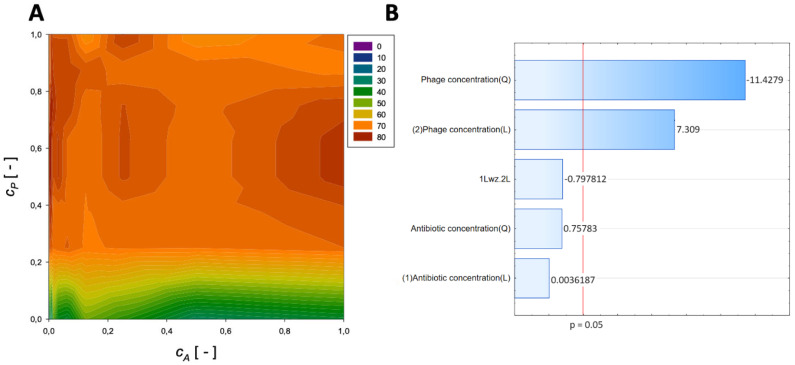
Surface response graphs of % reduction of biofilm biomass (**A**) and Pareto chart (**B**) of the synergistic effect of meropenem antibiotic and phage.

The obtained results are allowed to evaluate the concentrations of the various antibiotics (meropenem, amikacin, fosfomycin, ceftazidime, imipenem, gentamicin, and colistin) and the phage concentrations on the % reduction of biofilm biomass. The influence of meropenem and phage is graphically presented in Figure 1.

A second-order regression equation shown in Equation (4) was established to predict the % reduction via the application of meropenem and phage.

(4) $${Reduction}_{Meropenem}=54.7+73.1c_{A}-54.5c_{A}^{2}+37.5c_{P}-20.9c_{P}^{2}-12.6c_{A}^{}c_{P}^{},[\%]$$

Figure 1 shows that the % reduction is mainly affected by the application of phage. The obtained surface response graph (Figure 1A) was plotted to illustrate the mutual interactions between the independent variables. This graph can be used to determine the optimal conditions for the tested experimental system. Consequently, the results obtained through the surface graph made it possible to identify the best response when the most minor % reduction was considered the response variable.

The straight horizontal lines suggest that the antibiotic concentration and initial antibiotic concentration have minimal effect in the case of the system without the application of phage and for the usage of minimal phage concentration. The plot indicates that the % reduction increases with increasing phage concentration. Notably, the maximum % reduction for applying meropenem and phage is achieved at the normalized values of *c_A_* = 0.0625 and *c_P_* = 1, respectively. For these parameters, the % reduction is approximately 86%. These results indicate that biofilm biomass can be effectively reduced using phage with minimal application of antibiotics.

In the Pareto charts, the parameters that cross the reference line at 0.05 represent the factors with the highest statistically significant influence. The response surface was derived from the obtained models, and the Pareto ranking was performed. This analysis indicated that the most critical variable affecting the response was the phage concentration (linear and quadratic effects).

**Figure 2:**
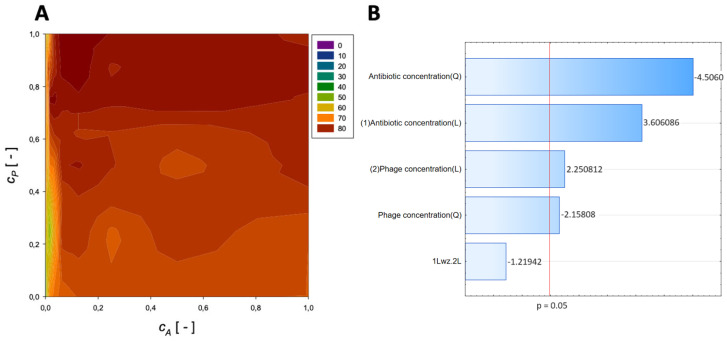
Surface response graphs of % reduction of biofilm biomass (**A**) and Pareto chart (**B**) of the synergistic effect of amikacin and phage.

A second-order regression equation shown in Equation (5) was established to predict the % reduction by applying amikacin and phage (the graphical presentation of this equation is presented in Figure 2).

(5) $${Reduction}_{Amikacin}=40.2+4.15c_{A}-7.5c_{A}^{2}+122.3c_{P}-91.4c_{P}^{2}-6.8c_{A}^{}c_{P}^{},[\%]$$

The maximum biofilm biomass reduction point was identified at a normalized antibiotic concentration equal to 0.00195 and a normalized phage concentration equal to 0.38. Biofilm biomass reduction reached 84.18%. These results indicate that biofilm biomass can be effectively reduced using phages with a minimal concentration of amikacin.

In the Pareto charts, the analysis indicated that the most critical variable affecting the response was antibiotic concentration (linear and quadratic effects), and phage concentration (both linear and quadratic effects) was statistically significant but to a lesser extent.

**Figure 3:**
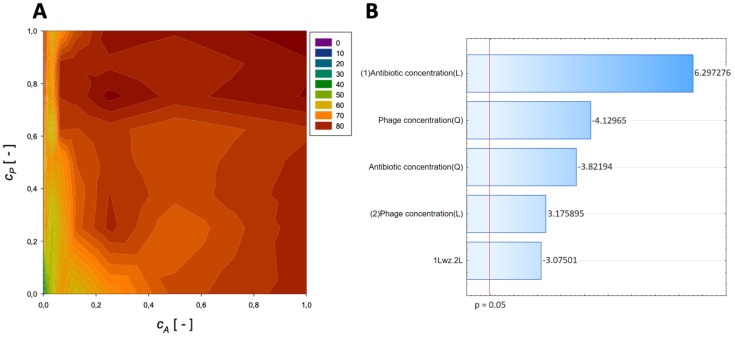
Surface response graphs of % reduction of biofilm biomass (**A**) and Pareto chart (**B**) of the synergistic effect of fosfomycin and phage.

A second-order regression equation shown in Equation (6) was established to predict the % reduction by applying phage (Figure 3 shows the effect of phage and antibiotics concentrations on % reduction of biofilm of biomass).

(6) $${Reduction}_{Fosfomycin}=44.8+71.6c_{A}-39.7c_{A}^{2}+60.5c_{P}-34.4c_{P}^{2}-27.2c_{A}^{}c_{P}^{},[\%]$$

The maximum biofilm biomass reduction point was identified at a normalized Fosfomycin concentration equal to 1 and a normalized phage concentration equal to 1. Biofilm biomass reduction reached 85.52%. These results indicate that biofilm biomass can be effectively reduced using a high concentration of phages combined with a high concentration of Fosfomycin.

The analysis indicated that the most important variable affecting the response was the linear effect of antibiotic concentration and the quadratic effect of phage concentration (Figure 3B). Also, other effects were statistically significant.

**Figure 4:**
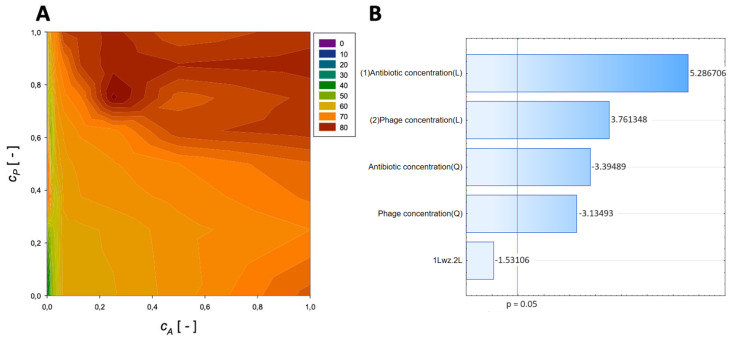
Surface response graphs of % reduction of biofilm biomass (**A**) and Pareto chart (**B**) of the synergistic effect of ceftazidime and phage.

A second-order regression equation shown in Equation (7) was established to predict the % reduction by applying ceftazidime and phage (the surface response described by this relation is shown in Figure 4).

(7) $${Reduction}_{Ceftazidime}=43.3+60.3c_{A}-36.9c_{A}^{2}+49.9c_{P}-27.4c_{P}^{2}-14.2c_{A}^{}c_{P}^{},[\%]$$

The maximum biofilm biomass reduction point was identified at a normalized ceftazidime concentration equal to 0.25 and a normalized phage concentration equal to 0.75. Biofilm biomass reduction reached 84.45%, and the obtained data indicate that biofilm biomass can be effectively reduced by using phages combined with ceftazidime.

In the Pareto charts, the analysis indicated that the most important variable affecting the response was the linear effect of the antibiotic concentration and phage concentration. The quadratic effect of antibiotic concentration and phage concentration was statistically significant.

**Figure 5:**
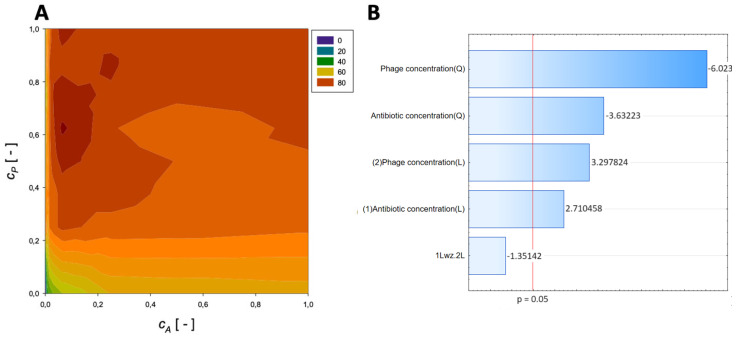
Surface response graphs of % reduction of biofilm biomass (**A**) and Pareto chart (**B**) of the synergistic effect of imipenem and phage.

A second-order regression equation shown in Equation (8) was established to predict the % reduction by applying imipenem and phage (Figure 5 presents the response surface for this case).

(8) $${Reduction}_{Imipenem}=39.7+75.9c_{A}-55.5c_{A}^{2}+101.7c_{P}-73.9c_{P}^{2}-17.6c_{A}^{}c_{P}^{},[\%]$$

The maximum biofilm biomass reduction point was identified at a normalized Imipenem concentration equal to 0.0625 and a normalized phage concentration equal to 0.63. Biofilm biomass reduction reached 88.74%, and the obtained data indicate that biofilm biomass can be effectively reduced by using phages combined with Imipenem.

In the Pareto charts, the analysis indicated that the most important variable affecting the response was the quadratic effect of the antibiotic concentration and phage concentration. The linear effect of antibiotic concentration and phage concentration was statistically significant.

**Figure 6:**
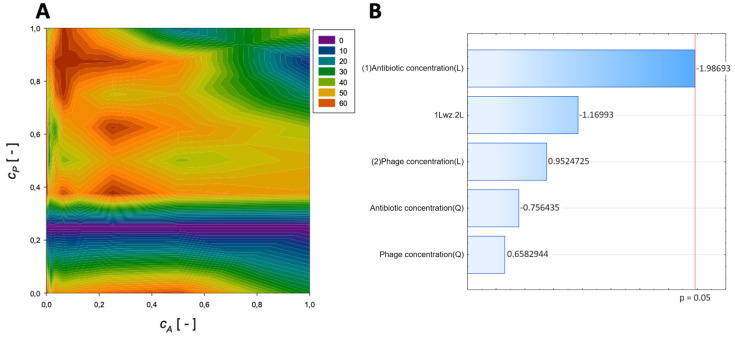
Surface response graphs of % reduction of biofilm biomass (**A**) and Pareto chart (**B**) of the synergistic effect of gentamicin and phage.

A second-order regression equation shown in Equation (9) was established to predict the % reduction by applying gentamicin and phage (the surface response described by this relation is presented in Figure 6).

(9) $${Reduction}_{Gentamicin}=37.3+18.7c_{A}-20.3c_{A}^{2}+8.9c_{P}-14.2c_{P}^{2}-26.8c_{A}^{}c_{P}^{},[\%]$$

The maximum biofilm biomass reduction point was identified at a normalized Gentamicin concentration equal to 0 and a normalized phage concentration equal to 1. Biofilm biomass reduction reached 68.36%, and the obtained data indicate that combining phage with gentamicin antagonism in action could decrease antibiofilm effectiveness in biofilm biomass reduction. In the Pareto charts, the analysis indicated that none of the used agents affected the response.

**Figure 7:**
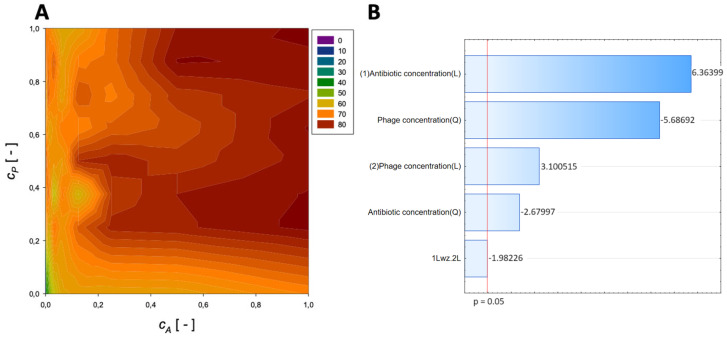
Surface response graphs of % reduction of biofilm biomass (**A**) and Pareto chart (**B**) of the synergistic effect of colistin and phage.

A second-order regression equation shown in Equation (10) was established to predict the % reduction by applying colistin and phage (Figure 7 presents the influence of phage and antibiotic concentrations on the % reduction of biofilm biomass).

(10) $${Reduction}_{Colistin}=45.5+55.1c_{A}-27.8c_{A}^{2}+68.3c_{P}-47.4c_{P}^{2}-17.6c_{A}^{}c_{P}^{},[\%]$$

The maximum biofilm biomass reduction point was identified at a normalized ceftazidime concentration equal to 1 and a normalized phage concentration equal to 0.25. Biofilm biomass reduction reached 84.45%, and the obtained data indicate that biofilm biomass can be effectively reduced using phages combined with colistin.

In the Pareto charts, the analysis indicated that the most important variable affecting the response was the linear effect of the antibiotic concentration and the quadratic effect of phage concentration. Also, the quadratic effect of antibiotic concentration and linear effect of phage concentration was statistically significant.

The highest effectiveness of the phage–antibiotic combination was observed for the imipenem–phage combination with a maximum reduction point ranging 88.74%. All top reduction points with specified normalized data are collected in Table 2.

In most cases, the significant variable influencing the response was the concentration of phages, emphasizing their crucial role in biofilm reduction. The combination of meropenem and phage exhibited the highest reduction (approximately 86%) at specific concentrations. Amikacin and phage were reduced by 84.18%, while phage reached 85.52% reduction when used at high concentrations. Ceftazidime and phage achieved an 84.45% reduction, and the imipenem–phage combination showed the highest effectiveness at 88.74%. However, gentamicin and phage exhibited antagonistic effects, resulting in a 68.36% reduction. Colistin and phage achieved an 84.45% reduction. The Pareto charts indicated that the importance of antibiotic and phage concentrations varied across different combinations. The data suggest that phage–antibiotic combinations can effectively reduce biofilm biomass, with the imipenem–phage combination demonstrating the highest efficacy. Still, the lower concentrations characterized by a high maximum reduction of biofilm biomass suggest phage amikacin combination at *c_A_* = 0.00195 and *c_P_* = 0.38 as the option that required minimum resources.

## 4. Discussion

Bacterial biofilm poses a severe threat due to its many times greater antibiotic tolerance. Bacteria in the form of biofilm pose a higher resistance to antibiotics. Antibiofilm activity of bacteriophages was reported before [30,31,32,33]. But, due to the increased interest in phage antibiotics, combination against biofilm, novel methods for accurate description, and fact optimization are needed. In this study, the RSM approach was utilized to determine the optimal points for the synergistic effect of antibiotic and phage concentrations on the % reduction of the biofilm biomass.

Response Surface Methodology has emerged as a crucial tool in biofilm research, enabling the optimization of growth conditions and biofilm formation for pathogenic microorganisms. This innovative approach is particularly evident in studies involving *Candida albicans* and *Escherichia coli* biofilm formation on medical prosthetics. In these investigations, RSM is employed to refine growth media components via Central Composite Design (CCD) models. This methodology considers multiple variables simultaneously, such as media pH, temperature, incubation period, shaker speed, and inoculum size, resulting in a comprehensive understanding of the intricate biofilm-forming processes. The conventional one variable at a time (OVAT) method needs to be revised in comparison. The quantification of biofilm is achieved using diverse techniques, including XTT assay for cell viability, crystal violet assay for biofilm matrix, and wet/dry weight measurements for cell mass. Notably, this study reveals the significance of factors like pH and fetal bovine serum in influencing biofilm formation. The application of RSM in optimizing *C. albicans* growth and biofilm formation offers a promising approach for future research in screening therapeutic interventions and advancing the control of biofilm-related challenges. This novel method significantly enhances our understanding of pathogenic biofilm interactions and paves the way for effective biofilm management strategies [34,35].

In this case, the primary objective of using RSM is to develop a mathematical model and analyze the interaction between independent parameters in the experiment. This model helps optimize the response surface and gain insights into how this surface varies concerning the independent variables of the tested process. A second-order model (Equation (1)) is employed in the RSM to achieve this. This model includes the process variables’ linear, second-order, and cross-product terms. This mathematical description enables the identification of local minima in the predicted response. Additionally, the most significant factors and interaction effects are detected using the Pareto plot [36,37,38]. The Pareto plot provides a clear visualization of which effects (phage concentration or antibiotics concentration) and two-factor interactions (between phage concentration and antibiotics concentration) are statistically significant at a 0.05 significance level. This graphical presentation is strongly readable.

This is the first study that reports the use of Response Surface Methodology utilization for the analysis of the antibiofilm interaction of bacteriophages and antibiotics. The observed phenomenon of synergistic eradication of biofilm, particularly when antibiotics are involved, can be elucidated by delving into the mode of action of the utilized medications. Specifically, the efficacy of ß-lactams and quinolones, which are cell wall synthesis inhibitors, can be attributed to their impact on virulent phage proliferation through mechanisms linked to cellular filamentation [7]. This present study also showed an increase in -lactams and quinolones used. When exposed to sub-lethal concentrations of antibiotics that disrupt cell wall integrity, bacterial cells trigger a stress response, consequently promoting enhanced replication of bacteriophages and subsequent cell lysis [7,39]. Notably, using Ciprofloxacin, Meropenem, and Minocycline has significantly reduced mortality among *Galleria mellonella* larvae infected with *Burkholderia cenocepacia*. This reduction can be attributed to the stimulation of phage lytic activity [9,40].

The synergistic action of Colistin and bacteriophages was observed before in planktonic forms of Acinetobacter baumannii by Wang et al. (2021). Our study indicates activity in biofilm, but the mode of action could be identical. The bacteriophage-mediated changes in envelope architecture, related to enzymatic activity, resensitizes *A.baumannii* to colistin. Additionally, Friunaviruses are characterized by coding exopolysaccharidases that could increase the penetration of antibiotics into biofilm [41,42,43,44]. Similar findings were also found for other bacteria like *Pseudomonas aeruginosa* and *Citrobacter amalonaticus* [45,46].

This present study found no synergistic action between phages and Gentamicin; instead, we observed an antagonistic interaction between gentamicin and bacteriophages. Biofilm biomass reduction point was identified at a normalized gentamicin concentration equal to 0 and a normalized phage concentration equal to 1, which means that bacteriophage alone reduces biofilm more effectively than combined with antibiotics. Kever et al. (2022) revealed significant antiphage properties of aminoglycosides. Molecular versatility of aminoglycosides, a primary class of translation-targeting antibiotics produced by *Streptomyces*, which have adapted to proficiently impede protein synthesis in bacterial rivals while concurrently furnishing protection against phage predation [47]. The previous study also showed that gentamicin decreases the effectiveness of bacteriophage vB_AbaP_AGC01 in the liquid culture medium [26]. Based on anti-biofilm activity, the results obtained in this work show that gentamicin, in the case of bacteriophage AGC01, is not the best choice because its effectiveness is reduced.

In summary, this study successfully demonstrated the synergistic effects of various antibiotics and phage concentrations on reducing biofilm biomass. The response surface graphs provided insights into the optimal conditions for achieving maximum reduction, while the Pareto charts helped identify the most influential variables. The findings underscored the importance of phage concentration in these interactions and provided valuable insights for designing effective strategies to combat biofilm-associated issues. Due to the specificity of phage–antibiotic interaction, an accurate description methodology of the observed effects is needed [3,12].

## 5. Conclusions

This is the first study that reports the use of Response Surface Methodology utilization for the analysis of the antibiofilm interaction of bacteriophages and antibiotics. Utilization of this methodology could provide fast analysis of bacteriophage-antibiotics assays. The results suggest that biofilm-mediated bacterial infections might be treated effectively with antibiotics and bacteriophages. In conclusion, our study used the RSM methodology to present antibiofilm PAS effectiveness mathematically. The results showed that this approach might determine the optimal conditions to enhance their combined antibiofilm activity. As a collection of statistical and mathematical tools for designing experiments and optimizing process parameters, RSM could be used to analyze bacteriophage-antibiotics interaction.

## Figures and Tables

**Table 2:** Points at which the maximum reduction of biofilm biomass is achieved.

Antibiotic	Normalized Antibiotic Concentration, *c_A_* [-]	Normalized Phage Concentration, *c_P_* [-]	Maximum Reduction [%]
Amikacin	0.00195	0.38	84.18
Meropenem	0.0625	1	85.79
Fosfomycin	1	1	85.52
Ceftazidime	0.25	0.75	84.45
Imipenem	0.0625	0.63	88.74
Gentamicin	0	1	68.36
Colistin	1	0.25	84.72

## Data Availability

The data presented in this study are available on request from the corresponding author.
